# Description of invasive aspergillosis in Mexican patients with chronic granulomatous disease

**DOI:** 10.70962/jhi.20250010

**Published:** 2025-07-18

**Authors:** Tiareth Cova-Guzmán, Deborah Palacios Reyes, Carla M. Román-Montes, Marco Antonio Yamazaki-Nakashimada, Aidé Tamara Staines Boone, Luis Silva-Goytia, María de la Luz García-Cruz, Héctor Gómez-Tello, Uriel Pérez-Blanco, Nancy Jiménez-Polvo, Estefany Mamani Velasquez, Nideshda Ramírez Uribe, Isabel Medina Vera, Sara Espinosa Padilla, Lizbeth Blancas-Galicia

**Affiliations:** 1 Laboratory of Immunodeficiency, National Institute of Pediatrics, Mexico City, Mexico; 2 Division of the Infectious Diseases, Mycology Department, National Institute of Pediatrics, Mexico City, Mexico; 3 Clinical Microbiology Laboratory, Instituto Nacional de Ciencias Médicas y Nutrición Salvador Zubirán, Mexico City, Mexico; 4 Department of Infectious Diseases, Instituto Nacional de Ciencias Médicas y Nutrición Salvador Zubirán, Mexico City, Mexico; 5 Department of Clinical Immunology, National Institute of Pediatrics, Mexico City, Mexico; 6 Department of Clinical Immunology, UMAE #25, IMSS, Monterrey, Mexico; 7 Department of Otorhinolaryngology, Instituto Nacional de Enfermedades Respiratorias, Mexico City, Mexico; 8 Department of Immunology, Poblano Children’s Hospital, Puebla, Mexico; 9 Department of Immunology, Children’s Hospital of Tlaxcala, Tlaxcala, Mexico; 10 Department of Pediatrics, Hospital del Niño “Dr. Ovidio Aliaga Uría”, La Paz, Bolivia; 11 Bone Marrow Transplant and Cell Therapy Program, National Institute of Pediatrics, Mexico City, Mexico; 12 Department of Research Methodology, National Institute of Pediatrics, Mexico City, Mexico

## Abstract

In chronic granulomatous disease (CGD), *Aspergillus* is the most common cause of invasive fungal infections and accounts for a high percentage of mortality. We recruited 45 patients of CGD with invasive aspergillosis (IA) events. The median age between the first CGD manifestation and first aspergillosis event was 65 mo. Mortality rate was 57.7% for proven aspergillosis events, 23.1% for probable events, and 19.2% for possible events (p = 0.038). *Aspergillus fumigatus* was the most common species. Comparing mortality with this cutoff point, 36% of those receiving <75 days of antifungal treatment died compared with 16.7% of those receiving >75 days (p = 0.05). 26 (58%) of the 45 patients died, out of which 18 (69%) had IA. Overall survival of the 45 patients was 80.8% at 64 mo. The high mortality rate of IA in CGD patients could be reduced by early suspicion, initiating correct antifungal treatment over a long period, and considering the performance of hematopoietic stem cell transplantation.

## Teaser

This study focuses on the clinical and microbiologic description of invasive aspergillosis in Mexican patients with chronic granulomatous disease. It highlights the diagnostic and therapeutic difficulties encountered and suggests the development of strategies to improve the outcome of these patients.

## Introduction

Chronic granulomatous disease (CGD) is a congenital immune disorder of phagocytic function caused by a defect in nicotinamide adenine dinucleotide phosphate (NADPH) oxidase (1). Patients with CGD lack reactive oxygen species production, which predisposes them to severe bacterial and fungal infections along with various inflammatory manifestations. Genes associated with classical CGD include NADPH oxidase 2 (*CYBB*), cytochrome B-245 α chain (*CYBA*), neutrophil cytosolic factor 1 (*NCF1*), and *NCF2*. *CYBB* has an X-linked (XL) inheritance pattern, whereas the other genes are inherited in an autosomal recessive pattern (1). Inherited p40^*phox*^ (*NCF4*) and chaperone EROS (*CYBC1*) deficiencies indicate an underlying distinctive condition resembling a mild, atypical form of CGD (2, 3).

Among the most common pathogens that infect patients with classical CGD include *Staphylococcus aureus*, *Aspergillus* spp., and *Burkholderia* spp. (4). *Aspergillus* infections have only been described in patients with pathogenic *CYBC1* variants and not in those with *NCF4* variants (2, 5, 6). Invasive *Aspergillus* infections are the most common fungal infections associated with CGD (4, 7, 8, 9, 10). Moreover, *Aspergillus fumigatus* is the most common pathogenic species, followed by *Aspergillus nidulans* (11). The clinical presentation of invasive aspergillosis (IA) in CGD is often indolent with minimal symptoms. Therefore, it is important to maintain a high index of clinical suspicion (8). The primary sites of IA in most patients are the lungs, followed by bones (8). These patients require prolonged and even multiple antifungal treatments (12). Stem cell transplantation (SCT) is a successful treatment for CDG and is an alternative for refractory IA (13). However, information on aspergillosis infections in patients with CGD is limited in Latin America (14, 15). Therefore, we aimed to describe the clinical, microbiological, and therapeutic characteristics of patients with CGD and IA in a Latin American country.

## Results

### Demographic and genetic description

The current study included 45 patients with a genetic diagnosis of CGD recruited between 2005 and 2024 from seven public hospitals in four Mexican cities (Mexico City, Monterrey, Tlaxcala, and Puebla). Tirthy (40%) of the 75 patients in this cohort were not included because they did not have aspergillosis at the time of the study. All subjects in this study were born in Mexico and continued to live there. The subjects were recruited from 44 unrelated families hailing from 15 of the 32 states in Mexico. All patients had infections other than IA and inflammatory manifestations (granulomas in different organs, autoimmunity, macrophage activation syndrome, inflammatory bowel disease, or Kawasaki disease). Three patients exhibited additional risk factors, including exposure to mulch (P7), visits to caves (P7), and residence with his mother in a jail cell with unsanitary conditions (P31). Thirthy-nine patients (87%) included in this study were male. The median age at the first manifestation of CGD was 3 mo (1–6.5); only one patient (P31) presented with IA as the first manifestation of CGD. The median age at CGD genetic diagnosis was 25 mo (9–66.5). The median time between the first manifestation of CGD and the first episode of IA was 60 mo (26.5 days–126 mo). The median age of all patients at last follow-up was 133 mo (73–200.5), and the median age of patients alive at the time of the study was 154.5 mo (111–238.2). The genotypes identified were *CYBB* (XL) in 33 cases (73%) and recessive genes in 12 cases (27%), with the following distribution: *NCF2* in six, *CYBA* in three, and *NCF1* in three patients. Regarding prophylaxis, 44 patients (97%) received itraconazole and trimethoprim with sulfamethoxazole, whereas 39 patients (86%) received additionally recombinant interferon gamma. Adherence to these treatments remains unknown (Table 1). The data on 45 patients have been previously published (4, 16).

**Table 1.** Molecular and clinical features of 45 patients with chronic granulomatous disease and invasive aspergillosis in Mexico

Patient code (P)	Sex	Gene	Events	IA Classification (EORTC/MSGERC)	*Aspergillus* species	Treatment	Time of Treatment		Steroids	Parallel antituberculosis therapy to antifungal therapy	Organs involved	Co-infection to aspergillosis	Clinical course	Cause of death
							<175 days	>75 days						
P1^(4)^	M	*CYBB*	2	Possible	​	Voriconazole	*	​	●	​	Lung	*Enterococcus* * faecalis*, *Achromobacter xylosoxidans*, and *Neisseria subflava*	Dead	IA
				Probable	​	Voriconazole	​	*	●	​	Lung			
P2^(4)^	M	*CYBB*	1	Probable	​	Voriconazole and caspofungin	​	*	●	​	Lung	*Pseudomonas* * aeruginosa*	Alive	​
P3^(4)^	M	*CYBB*	4	Possible	​	Voriconazole, caspofungin, and amphotericin B	*	​	●	R, H, Z, and E	Lung, liver, spleen, and kidney	*Serratia* * marcescens*	Dead	Cardiogenic shock
				Possible	​	Voriconazole	​	*	​	​	Lung and bone			
				Possible	​	Voriconazole and caspofungin	​	*	​	​	Lung and bone			
				Possible	​	Voriconazole and fluconazole	*	​	●	​	Lung			
P4^(4)^	M	*NCF2*	1	Possible	​	Voriconazole and caspofungin	*	​	●	​	Lung	​	Dead	Septic shock
P5^(4)^	M	*CYBB*	4	Possible	​	Voriconazole	​	*	●	R, H, Z, and E	Lung	*Exophiala* * dermatitidis*	Alive	​
				Possible	​	Voriconazole	​	*	●	​	Lung			
				Probable	​	Voriconazole and amphotericin B	*	​	●	​	Lung			
				Probable	​	Voriconazole	​	*	●	​	Lung			
P6^(4)^	M	*CYBB*	2	Possible	​	Voriconazole and caspofungin	​	*	​	R, H, Z, E, and LVX	Lung	​	Alive	​
				Possible	​	Voriconazole	*	​	​	​	Lung			
P7^(4)^^a^	F	*NCF2*	2	Possible	​	Voriconazole and posaconazole	​	*	​	​	Lung and spleen	​	Alive	​
				Probable	*A. fumigatus*	Voriconazole	​	*	​	H, E, CLR, and LVX	Lung			
P8	M	*CYBB*	1	Probable	​	Voriconazole and caspofungin	*	​	●	H, R, E, CIP, and CLR	Lung	​	Dead	IA
P9^(4)^	F	*CYBA*	3	Probable	*Aspergillus* spp	Voriconazole	​	*	​	​	Lung	*Salmonella* spp., *P. aeruginosa*, *Citrobacter freundii*, *Klebsiella variicola*, and *Mycobacterium chimaera*	Dead	IA
				Probable	​	Voriconazole	​	*	●	​	Lung			
				Probable	*A. niger* *A. terreus*	Voriconazole, caspofungin, and amphotericin B	​	*	●	R, Z, E, and H	Lung			
P10	M	*CYBB*	1	Possible	​	Voriconazole, caspofungin, and posaconazole	​	*	●	LVX, H, Z, and E	Lung	​	Dead	Septic shock
P11^(4)^	M	*NCF2*	1	Probable	​	Voriconazole, caspofungin, and amphotericin B	*	​	●	H, R, and E	Lung, skin, and CNS	​	Dead	IA
P12^(4)^	M	*CYBB*	1	Proven	*A. versicolor*	Voriconazole	​	*	●	​	Lung, liver, spleen, and CNS	*S. marcescens* and *Burkholderia cepacia*	Alive	​
P13^(4)^	M	*CYBB*	2	Probable	​	Voriconazole, caspofungin, and amphotericin B	​	*	●	​	Lung and spleen	*Pneumocystis* * jirovecii*	Dead	IA
				Probable	*A. fumigatus*	Voriconazole	*	​	​	​	Lung			
P14^(4)^	F	*NCF2*	4	Possible	​	Voriconazole	​	*	●	CLR, R, H, and E	Lung	*E. coli BLEE*, *Klebsiella pneumoniae BLEE*, *P. aeruginosa*, *Pseudomonas rhodesiae*, *Streptococcus oralis*, *Streptococcus salivarius*, *Penicillium* spp., and *Campylobacter* spp.	Alive	​
				Possible	​	Voriconazole, caspofungin, and amphotericin B	​	*	●	​	Lung, liver, and spleen			
				Proven	*A. ustus*	Voriconazole	*	​	​	CLR, R, H, and E	Lung			
				Probable	​	Voriconazole	​	*	●	​	Lung			
P15^(4)^	M	*CYBB*	1	Probable	​	Voriconazole	​	*	●	​	Lung	​	Alive	​
P16	M	*CYBB*	1	Possible	​	Voriconazole	​	*	​	​	Lung	​	Dead	IA
P17^b^	M	*CYBB*	1	Probable	*A. terreus*	Voriconazole and caspofungin	*	​	●	​	Lung	*Actinomyces * *israelii* and *Staphylococcus aureus*	Dead	IA
					*A. flavus*									​
P18^(4)^	M	*CYBB*	2	Possible	​	Voriconazole	*	​	​	​	Lung	*Salmonella* spp.	Dead	IA
				Probable	*A. fumigatus*	Voriconazole, caspofungin, amphotericin B, and posaconazole	​	*	●	​	Lung and CNS			
P19^(4)^	M	*CYBB*	1	Probable	​	Voriconazole and caspofungin	*	​	●	​	Lung	​	Dead	IA
P20^(4)^	M	*CYBA*	3	Possible	​	Voriconazole	*	​	​	​	Lung	*S. marcescens*, *Burkholderia multivorans*, *P. aeruginosa*, and *K. pneumoniae BLEE*	Dead	Pneumonia *K*.* pneumoniae*
				Possible	​	Voriconazole	​	*	​	​	Lung and spleen			
				Proven	*A. flavus*	Voriconazole, caspofungin, and amphotericin B	​	*	●	​	Lung and CNS			
P21^(4)^	F	*NCF2*	2	Possible	​	Voriconazole and caspofungin	​	*	●	​	Lung	*Salmonella* spp., *Streptococcus pneumoniae*, *P. aureginosa*, and *Hormographiella aspergillata*	Alive	​
				Probable	​	Voriconazole	*	​	●	​	Lung			
P22^(4)^	M	*NCF1*	3	Possible	​	Voriconazole	*	​	●	​	Lung	*K. pneumoniae* and *Enterobacter cloacae*	Alive	​
				Possible	​	Voriconazole	​	*	​	H, E, LZD, and LVX	Lung			
				Probable	​	Voriconazole, caspofungin, and amphotericin B	​	*	​	H, E	Lung and CNS			
P23^(4)^	M	*CYBB*	1	Probable	​	Voriconazole and caspofungin	​	*	●	​	Lung	*E. coli*	Dead	Septic shock
P24	M	*CYBB*	1	Probable	*Aspergillus* spp	Voriconazole	*	​	●	​	Lung	*Staphylococcus* *hominis*	Alive	​
P25^(4)^	M	*CYBB*	1	Possible	​	Voriconazole, caspofungin, and amphotericin B	​	*	●	​	Lung	*Staphylococcus haemolyticus* and *Stenotrophomonas maltophilia*	Dead	IA
P26^(4)^	M	*NCF1*	3	Possible	​	Voriconazole	*	​	​	​	Lung	*Candida * *guilliermondii*	Alive	​
				Proven	*A. fumigatus*	Voriconazole	​	*	●	​	Lung			
				Proven	*A. nidulans* *A. fumigatus* *A. versicolor*	Voriconazole and amphotericin B	​	*	●	​	Lung			
P27^(24)^	M	*CYBB*	1	Probable	*Aspergillus* spp	Voriconazole, caspofungin, amphotericin B, and posaconazole	​	*	●	H, E, and LVX	Lung, skin, and CNS	​	Dead	IA
P28	M	*CYBB*	1	Probable	​	Voriconazole, caspofungin, and amphotericin	​	*	●	H, R, Z, and E	Lung	​	Alive	​
P29^(4)^	M	*CYBB*	3	Possible	​	Voriconazole	*	​	​	​	Lung	​	Alive	​
				Possible	​	Voriconazole	​	*	​	​	Lung			
				Possible	​	Voriconazole	​	*	●	​	Lung			
P30^(4)^	M	*CYBB*	1	Possible	​	Voriconazole and caspofungin	​	*	●	​	Lung	​	Alive	​
P31^(^^1^^6)^^c^	M	*CYBB*	2	Probable	*Aspergillus* spp	Voriconazole	*	​	​	​	Lung, bone, and skin	*E. faecalis*	Dead	IA
				Probable	*A. fumigatus*	Voriconazole	*	​	​	​	Lung			
P32^(4)^	M	*CYBB*	1	Proven	*Aspergillus* spp	None	​	​	●	​	Lung	*B. cepacia*	Dead	IA
P33	M	*CYBB*	1	Proven	*A. fumigatus*	Fluconazole	​	*	●	​	Lung	*Mycobacterium * *tuberculosis* and *K. pneumoniae*	Dead	IA
P34^(4)^	F	*CYBB*	1	Probable	​	Voriconazole and amphotericin B	*	​	●	​	Lung	​	Alive	​
P35^(4)^	M	*CYBB*	1	Proven	*A. versicolor*	Voriconazole	​	*	●	H, R, Z, and E	Lung	​	Alive	​
P36^(4)^	M	*CYBB*	2	Possible	​	Voriconazole	​	*	●	​	Lung and liver	​	Alive	​
				Probable	*A. fumigatus*	Voriconazole	​	*	●	​	Lung and skin			
P37^(4)^	M	*NCF2*	4	Possible	​	Voriconazole, caspofungin, and amphotericin B	​	*	​	​	Lung	*S. marcescens*	Alive	​
				Probable	​	Voriconazole and caspofungin	​	*	​	R, H, Z, and E	Lung			
				Proven	*A. versicolor*	Voriconazole	​	*	●	​	Lung			
				Proven	*A. fumigatus*	Voriconazole	​	*	●	​	Lung and skin			
P38^(4)^	F	*CYBA*	1	Probable	*A. fumigatus*	Voriconazole	*	​	​	​	Lung	​	Dead	Accident
P39	M	*CYBB*	2	Possible	​	Voriconazole	​	*	​	​	Lung	*Pseudomonas * *putida* and *K. pneumoniae BLEE*	Alive	​
				Probable	*A. versicolor*	Voriconazole	​	*	​	​	Lung			
P40	M	*CYBB*	1	Probable	​	Amphotericin B	*	​	●	​	Lung	*S. aureus* and *S. maltophilia*	Dead	Septic shock
P41	M	*CYBB*	1	Proven	*A. nidulans*	Voriconazole, caspofungin, and amphotericin B	*	​	●	​	Lung	*B. cepacia*, *Penicillium* spp., and *Pseudomonas* spp.	Dead	IA
P42	M	*CYBB*	1	Probable	*A. fumigatus* *A. versicolor*	Voriconazole	*	​	​	​	Lung and bone	*A. lwoffi* and *P. aeruginosa*	Dead	IA
P43	M	*CYBB*	1	Proven	*A. versicolor*	Voriconazole, caspofungin, amphotericin B, and posaconazole	​	*	●	​	Lung and pericardium	*S. aureus* and *S. hominis*	Dead	IA
P44	M	*CYBB*	1	Proven	*Aspergillus* spp	Voriconazole, caspofungin, and amphotericin B	​	*	●	​	Lung and bone	*Streptococcus * *mitis*, *K. pneumoniae*, *P. aeruginosa*, *S. hominis*, and *Candida albicans*	Dead	IA
P45^(4)^	M	*NCF1*	4	Possible	​	Voriconazole, itraconazole, and anidulafungin	*	​	​	​	Lung	​	Dead	Cerebral cryptococcosis
				Probable	*A. fumigatus* *A. flavus*	Itraconazole	​	*	●	​	Lung			
				Probable	*A. fumigatus*	Voriconazole, amphotericin B, itraconazole, fluconazole, and anidulafungin	*	​	​	​	Lung			
				Probable	*A. flavus*	Voriconazole, caspofungin, and amphotericin B	*	​	●	​	Lung			

M, Male; F, Female; H, isoniazid; R, rifampicin; E, ethambutol; Z, pyrazinamide; CLR, clarithromycin; LVX, levofloxacin; CIP, ciprofloxacin; LZD, linezolid. Superscript numbers in column one indicate bibliographic citations if patient previously published. * time of antifungal treatment; ● the patient received steroids.

a The patient was exposed to mulch.

b The patient visited a cave.

c The patient lived in jail after birth.

### Invasive aspergillosis

**Figure 1.**
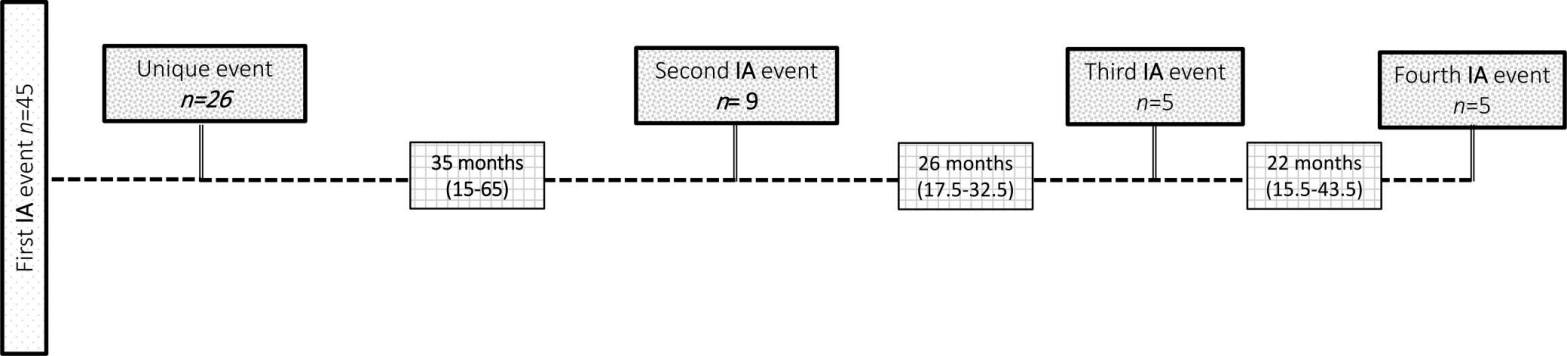
**Median time at the first invasive aspergillosis (IA) event and subsequent events.** Different boxes present the number (*n*) of patients with one, two, three, or four events. The median time is expressed in months with the range in parentheses.

Of the 44 families of the 45 patients, one family had two affected members with IA. The median age at the first IA event in all 45 patients was 65 mo (30.5–140). A total of 79 IA events were recorded in 45 patients. The difference in the median age at the first IA event between the 33 patients with XL inheritance (64 mo [30.5–140]) and the 12 patients with AR inheritance (85.5 mo [25.2–166.7]) was not significant (p = 0.586). Thirteen patients (29%) had at least one IA event before CGD diagnosis, median period time between the first IA event and the CGD diagnosis was 1 mo (1, 2, 3), and 32 patients (71%) had one or more IA events after CGD diagnosis, median period time between the CGD diagnosis and the first IA event was 46.5 mo (13.2–85.5). Of the 45 patients, 26 (58%) had one IA event, nine (20%) had two, five (11%) had three, and five (11%) had four IA events. The median time between the first and second events was 35 mo (15–65), that between the second and third events was 26 mo (17.5–32.5), and that between the third and fourth events was 22 mo (15.5–43.5) (Fig. 1).

### Microbiological features

**Figure 2.**
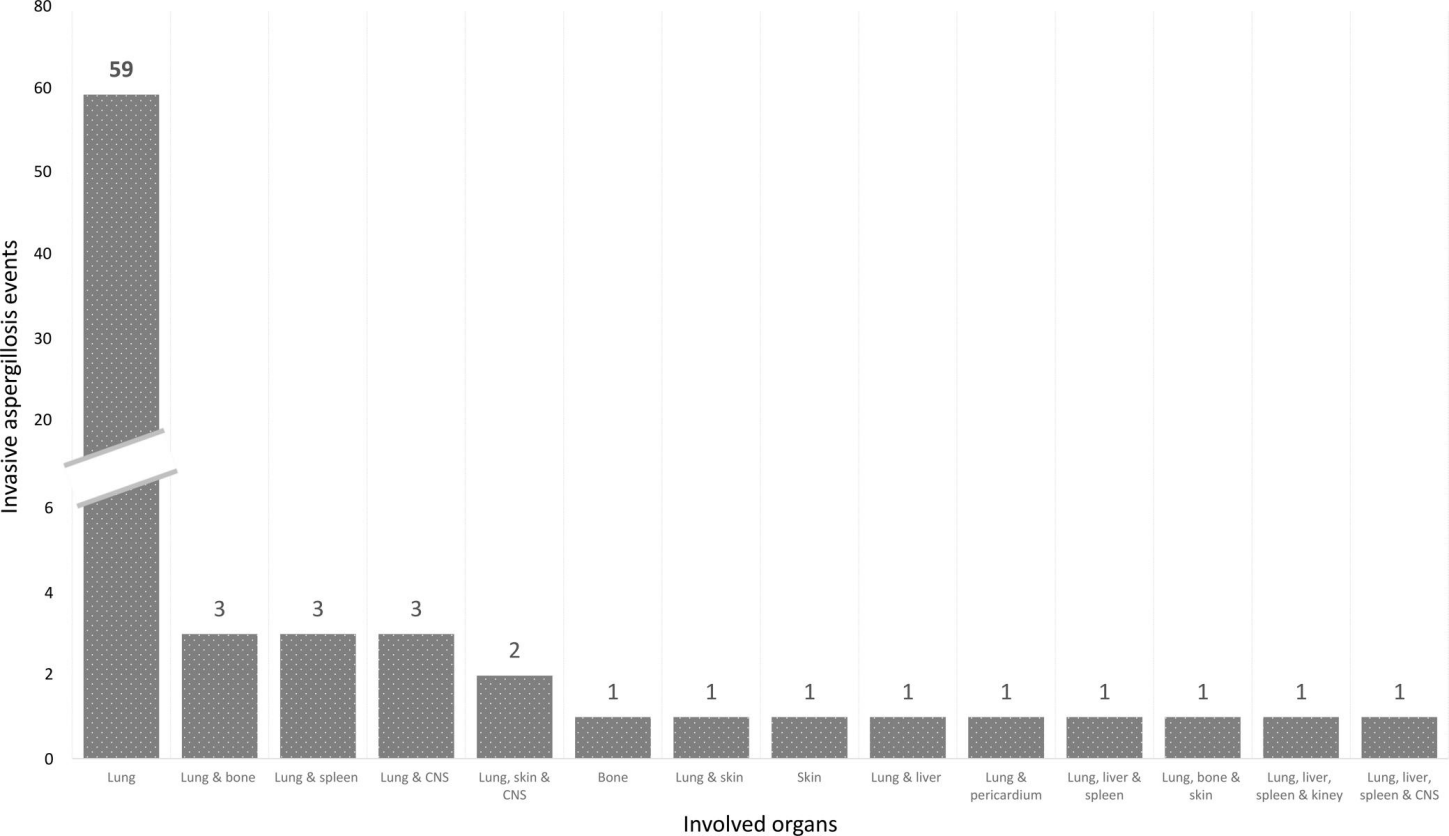
**Organ(s) involved in 79 events of invasive aspergillosis in 45 patients with chronic** **granulomatous disease** **.** X axis presents the involved organs, and Y axis presents the number or events.

Based on the European Organization for Research and Treatment of Cancer/the Mycoses Study Group Education and Research Consortium (EORTC/MSGERC) criteria (17), from the 79 events, 13 (17%) IA events were proven, 35 (44%) were probable, and 31 (39%) were possible (Table 1). The mortality rate was 57.7% for proven events, 23.1% for probable events, and 19.2% for possible events (p= 0.038). The median time between the onset of symptoms and the isolation of *Aspergillus* spp. was 35 days (19.5–55.5). *Aspergillus* spp. was detected in 30 events, 13 of which were *Aspergillus fumigatus* and the other 17 included *Aspergillus versicolor* (*n* = 7), *Aspergillus terreus* (*n* = 2), *Aspergillus nidulans* (*n* = 2), *Aspergillus flavus* (*n* = 4), *Aspergillus ustus* (*n* = *1*), and *Aspergillus niger* (*n* = 1) (Tables 2 and 3). The proportion of mortality was not significantly different between patients infected with one *Aspergillus* species (48%) and those infected with ≥2 *Aspergillus* species (60%) (p = 0.5). The median time from symptom onset to *Aspergillus* antigen (galactomannan) positivity was 21 days (9.7–59.7) among the 35 probable events. The 79 IA events affected the following organs: lungs (*n* = 59); lungs and bones (*n* = 3); lungs and spleen (*n* = 3); lungs and central nervous system (CNS; *n* = 3); lungs, skin, and CNS (*n* = 2); bones (*n* = 1); skin (*n* = 1); lungs, and skin (*n* = 1); lungs and liver (*n* = 1); lungs and pericardium (*n* = 1); lungs, livers, and spleen (*n* = 1); lungs, bones, and skin (*n* = 1); lungs, liver, spleen, and kidneys (*n* = 1); and lungs, liver, spleen, and CNS (*n* = 1) (Fig. 2). The proportion of mortality was not significantly different between patients with only pulmonary IA (32.2%) and IA pulmonary and one or more IA organs affected (35%) (p = 0.818). Co-infection with bacteria and fungi was not uncommon; the Table 1 shows the different microorganisms that co-infected each patient in each of the IA events (Table 1).

**Table 2.** Detected *Aspergillus* species in different sterile organs and body fluids in 13 different proven events in 11 patients

Event number	Code patient (P)	Species identified by culture	Detection site
1	P12	*A. versicolor*	Brain
2	P14	*A. ustus*	Lung
3	P20	*A. flavus*	Brain and lung
4	P26	*A. fumigatus*	Lung
5	P26	*A. nidulans*	Lung and pleural fluid
		*A. fumigatus*	
		*A. versicolor*	
6	P32	*Aspergillus* spp.	Lung
7	P33	*A. fumigatus*	Lung (autopsy)
8	P35	*A. versicolor*	Lung
9	P37	*A. versicolor*	Lung
10		*A. fumigatus*	Lung and skin
11	P41	*A. nidulans*	Lung
12	P43	*A. versicolor*	Pericardial fluid
13	P44	*Aspergillus* spp.	Lung and bone

In P26 there were three species, and in P37 there were two species.

**Table 3.** Detected *Aspergillus* species in non steril body fluids in 17 different events from 13 patients

Event number	Code patient (P)	Species identified by culture	Detection site
1	P7	*A. fumigatus*	BAL
2	P9	*Aspergillus* spp.	BAL
3		*A. niger* and *A. terreus*	BAL
4	P13	*A. fumigatus*	BAL
5	P17	*A. terreus* and *A. flavus*	BAL
6	P18	*A. fumigatus*	BAL
7	P24	*Aspergillus* spp.	BAL
8	P27	*Aspergillus* spp.	BAL, purulent discharge from an abscess
9	P31	*Aspergillus* spp.	Purulent discharge from an abscess
10		*A. fumigatus*	BAL
11	P36	*A. fumigatus*	BAL, purulent discharge from an abscess
12	P38	*A. fumigatus*	BAL
13	P39	*A. versicolor*	Blood
14	P42	*A. fumigatus* and *A. versicolor*	BAL, purulent discharge from an abscess
15	P45	*A. fumigatus* and *A. flavus*	BAL
16		*A. fumigatus*	BAL
17		*A. flavus*	BAL

P9 and P45 had two species. BAL, bronchoalveolar fluid.

### Antifungal therapy

Fourty-four (97%) patients with 78 events received antifungal therapy. The median time between hospital admission and initiation of antifungal treatment was 13.5 days (7–30). One patient (P32) did not receive treatment because IA was diagnosed postmortem. All 44 patients received one or more of the following antifungal agents: voriconazole, amphotericin B, caspofungin, posaconazole, itraconazole, or anidulafungin. Of the 78 IA events, 43 (55%) were treated with monotherapy, 16 (20.5%) with dual therapy, 15 (19.2%) with triple therapy, three (3.8%) with quadruple therapy, and one (1.5%) with quintuple therapy (Table 1). Patients who received more than two antifungal agents per event had a higher rate of mortality (48.6%) compared with patients who received only one antifungal agent (20.5%) (p = 0.008). We compared the number of days of antifungal treatment in the group of patients who died and those who survived; we found no statistically significant differences. Receiver-operating characteristic curve (ROC) curve analysis was performed to relate the number of treatment days to mortality, resulting in a cutoff point of <75 treatment days. Comparing mortality with this cutoff point, 36% of those receiving <75 days of treatment died versus 16.7% of those receiving >75 days (p = 0.05). Among patients coinfected with tuberculosis and aspergillosis there was no statistically significant difference (p = 0.516) in the proportion of deaths in patients receiving voriconazole plus antimycobacterial drugs, including rifampicin (27.3%) versus voriconazole plus other antimycobacterial drugs without rifampicin (40%). In 51 (65%) of the 78 events, parallel therapy with corticosteroids (methylprednisolone, prednisone, dexamethasone, or hydrocortisone) was administered; however, information on the exact indication is unavailable.

### Outcome

**Figure 3.**
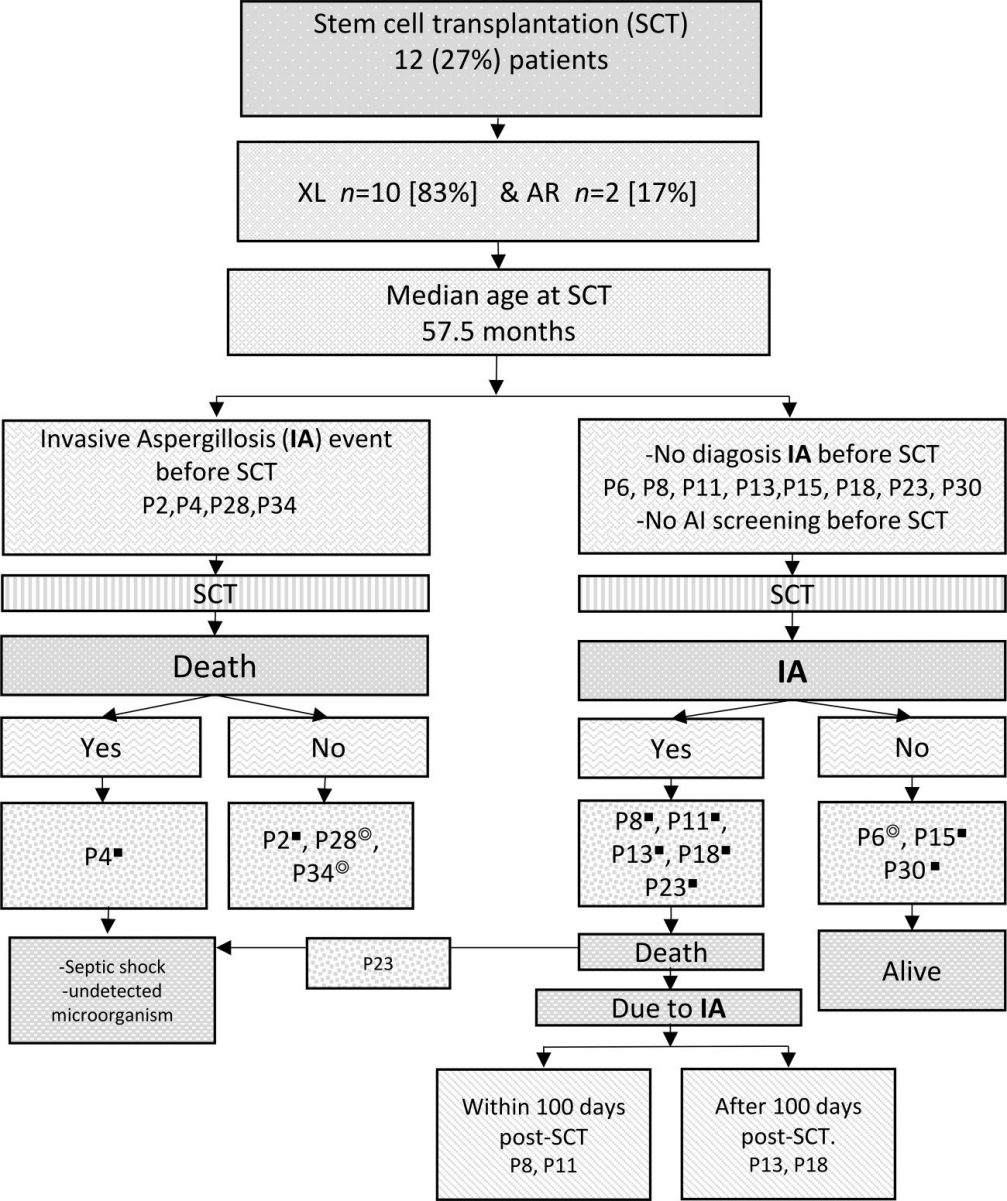
**Stem cell trasplantation (SCT) in 12 patients with chronic granulomatous disease.** Twelve of the 45 patients were subjected to transplantation, four of which had invasive aspergillosis (IA) before SCT and eight after. P, patients; AR, autosomal recessive. ◎Full myeloablative conditioning. ■ Reduced intensity conditioning.

Among the described patients, 12 (27%) underwent at least one SCT, and the median age at the time of transplantation was 57.5 mo (19.2–158.2). In the cohort under investigation, nine patient received reduced intensity and three myeloablative conditioning. Among the 12 transplanted patients, those receiving complete myeloablative conditioning reported no deaths and those receiving reduced intensity six deaths (66.7%) (p = 0.091). Four out of 12 patients experienced an IA event prior to undergoing SCT. Of these patients, three underwent successful grafting, while one patient died owing to septic shock (microorganism undetected) (Fig. 3). The remaining eight patients were not evaluated purposely for aspergillosis before the procedure. Five out of the eight cases died; one as a result of septic shock (microorganism undetected) and four as a result of IA during the procedure, while the remaining three had a successful graft. In the four patients with IA after SCT, the median time between the procedure and the diagnosis of IA was 21 days (7–145).

**Figure 4.**
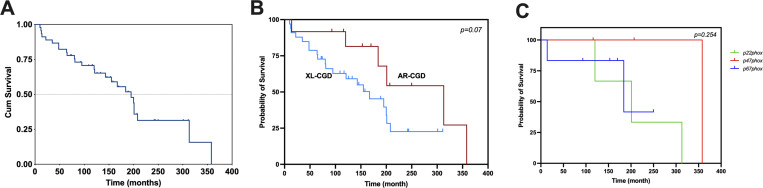
**Survival in 45 patients with crhonic granulomatous disease and invasive aspergillosis. (A)** Overall survival of the 45 patients was 67.9% at 120 mo. **(B)** Median survival time for patients with XL-CGD was 167 mo and it was more than 313 mo for patients with AR-CGD (p = 0.07). **(C)** When survival analysis of the AR-CGD subgroups was performed, no significant differences were observed (p = 0.254).

Twenty-six of the 45 patients died, 18 (69%) from IA and the remaining from other causes (Table 4). Sixteen (89%) of the deceased patients had XL inheritance, while the rest had AR inheritance (p = 0.054). The overall survival of the 45 patients was 80.8% at 64 mo and 67.9% at 120 mo, with a median survival of 195 mo. The median survival time for patients with XL-CGD was 167 mo, with 20 deaths, and for patients with AR-CGD it was more than 313 mo, with 6 deaths (p = 0.07). When survival analysis of the AR-CGD subgroups was performed, no significant differences were observed (p = 0.254), median survival for the p22^*phox*^ group was 201 mo with three deaths, for the p47^*phox*^ group 358 mo with one death, and finally, for the p67^*phox*^ group 184 mo with two deaths (Fig. 4). The median age at death was 107.5 mo (44.7–196), and the median time between the first IA event and death was 6 mo (1–15). *Aspergillus* spp. was isolated from 10 (55%) of the 18 patients who died of IA; the species are listed in Table 1.

**Table 4.** The clinical and microbiological profile of 18 patients with chronic granulomatous disease who died secondary to invasive aspergillosis

​	Patient code (P)	Affected organs	*Aspergillus *detection	Treatment	Co-infections	Age of death
1	P1	Lung	Galactomannan antigen	Voriconazole	*Achromobacter * *xylosoxidans* and *N. subflava*	16 yo 9 mo
2	P8	Lung	Galactomannan antigen	Voriconazole and caspofungin	​	1 yo 10 mo
3	P9	Lung	*A. niger*	Voriconazole, caspofungin, and amphotericin B	*Salmonella* spp, *P. aeruginosa*, *C. freundii*, *K. variicola*, and *M. chimaera*	16 yo 9 mo
			*A. terreus*			
4	P11	Lung, skin, and CNS	Galactomannan antigen	Voriconazole, caspofungin, and amphotericin B	None	15 yo 4 mo
5	P13	Lung	*A. fumigatus*	Voriconazole	*P. jirovecii*	4 yo
6	P16	Lung	Clinical and radiological findings	Voriconazole	None	11 yo 11 mo
7	P17	Lung	*A. terreus*	Voriconazole and caspofungin	*A. israelii* and *S. aureus*	5 yo 5 mo
			*A. flavus*			
8	P18	Lung and CNS	*A. fumigatus*	Voriconazole, caspofungin, amphotericin B, and posaconazole	*Salmonella* spp.	7 yo 11 mo
9	P19	Lung	Galactomannan antigen	Voriconazole and caspofungin	None	1 yo 1 mo
10	P25	Lung	Clinical and radiological findings	Voriconazole, caspofungin, and amphotericin B	*S. haemolyticus* and *S. maltophilia*	6 yo 8 mo
11	P27	Lung, skin, and CNS	*Aspergillus* spp.	Voriconazole, caspofungin, amphotericin B, and posaconazole	None	4 yo
12	P31	Lung	*A. fumigatus*	Voriconazole	None	12 mo
13	P32	Lung	*Aspergillus* spp.	None	*B. cepacia*	10 mo
14	P33	Lung	*A. fumigatus*	Fluconazole	*M. tuberculosis* and *K. pneumoniae*	2 yo 11 mo
15	P41	Lung	*A. nidulans*	Voriconazole, caspofungin, and amphotericin B	*B. cepacia*, *Penicilium* spp., and *Pseudomonas* spp.	12 yo 11 mo
16	P42	Lung and bone	*A. fumigatus* and *A. versicolor*	Voriconazole	*A. lwoffi* and *P. aeruginosa*	16 yo 16 mo
17	P43	Lung and pericardium	*A. versicolor*	Voriconazole, caspofungin, amphotericin B, and posaconazole	*S. aureus* and *S. hominis*	10 yo 1 mo
18	P44	Lung and bone	*Aspergillus* spp.	Voriconazole, caspofungin, and amphotericin B	*S. mitis*, *K. pneumoniae*, *P. aeruginosa*, *S. hominis*, and *C. albicans*	6 yo 9 mo

yo, years old.

## Discussion

This is the first study on patients with CGD and IA in a Latin American country. The 45 patients included in this study were treated in public hospitals, with the majority coming from low socioeconomic backgrounds. All patients in the present study had pathogenic variants of the four genes involved in classical CGD (*CYBB*, *CYBA*, *NCF1*, and *NCF2*) (18). In a cohort of 24 North American patients with CGD receiving prophylactic itraconazole, the first event of invasive fungal infection occurred at the age of 10 years compared with 4 years in patients that did not receive itraconazole (12). Most of the patients described here experienced their first IA event before the age of 5 years, and although they were prescribed prophylactic itraconazole, we have no knowledge of their treatment adherence and compliance. Routine monitoring of itraconazole plasma levels (target level of >250 μg/ml for prophylaxis) is mandatory as a method to assess adherence and compliance; levels must be monitored every 3 mo in patients with CGD that are not transplanted (19).

Each event was classified according to the EORTC/MSGERC criteria, with 13 events classified as proven, 35 as probable, and 31 as possible. The proven events had a higher mortality; a possible explanation is that the other two groups had a lower infectious burden or were actually free of aspergillosis. Similar to other studies, we found that in few patients with proven IA events, the previous results for serum galactomannan antigen were negative (12, 13). Blumental *et al.* reported that the lack of sensitivity can be attributed to the absence of angioinvasion by fungal hyphae observed during IA in patients with CGD, in contrast to neutropenic subjects (12). In addition, fungal elements have been identified in lung biopsies with no growth in culture (20). Therefore, a negative test result in patients with CGD does not rule out the possibility of aspergillosis. Similar to in other patient groups, *A. fumigatus* was the most common species to infect the 45 Mexican patients (7, 21, 22, 23, 24, 25, 26). Other species identified in our study included *A*. *nidulans*, *A. terreus*, *A*. *versicolor*, *A*. *flavus*, *A*. *ustus*, and *A. niger*. Interestingly, there was no increased mortality in patients infected with two species compared with those infected with one species. To the best of our knowledge, *A*. *ustus* (P14) has not been previously described in patients with CGD. *A. ustus* has been found on the surfaces of the walls of caves, in the indoor air of buildings, including hospitals, and in soils and bat dung (27). A comparison of our results with other descriptions of CGD and IA confirmed that *Aspergillus* primarily affected the lungs, followed by other anatomical sites, such as the bones, CNS, skin, and spleen (7, 10, 21, 25, 28). In this study, we found no increased mortality in patients with pulmonary IA versus those with pulmonary and other organ involvement. Pericardial involvement of *Aspergillus* was found in one patient (P43). A similar case was reported by Abd Elaziz *et al.* (25). Furthermore, one patient (P3) had IA involving the kidneys, which is the first reported case in literature involving this organ. We found that after the first IA event, subsequent aspergillosis events occurred, and the time interval between the following IA events decreased (Fig. 1).

According to Kline *et al.*, single-agent therapy for IA often provides unsatisfactory results; however, the benefits of combined therapy are debated (13). In the 78 patients, voriconazole monotherapy was the first-line treatment for most patients. However, several patients experienced subsequent IA events. In this study, patients who received more than two antifungals per event had a higher mortality rate than those who received a single antifungal; one explanation could be that by the time an additional antifungal was administered, the aspergillosis had spread and was refractory despite polytherapy. Combination antifungal therapy starting from the first episode of aspergillosis may be effective in preventing subsequent episodes. Among the 45 patients who received <75 days of treatment versus those who received >75 days had higher mortality. The neonatal patient (P31) was administered voriconazole monotherapy for 28 days with apparent improvement; however, a few months later, he presented with a second fatal event. Prolonged administration of multiple antifungals is necessary to achieve remission of infection (12). Blumental *et al*. suggested ∼446 days of treatment for cure (12).

Patients with inborn errors of immunity are at risk of exposure to endemic microorganisms depending on their geographical region of residence. Mycobacterial infections are widespread among patients with CGD in Latin America (13). These patients are often co-infected with fungi and mycobacteria in tuberculosis endemic areas, which require treatment using antifungals in addition to antituberculosis drugs (Table 1) (29). In such cases, it is crucial to note that combining rifampicin with voriconazole treatment leads to a significant reduction in the plasma concentration of the antifungal drug (30), thereby compromising treatment efficacy. Alternative medications should be considered in such cases. *Aspergillus* infection triggers an abnormal immune response in patients with CGD. This response is characterized by hyperinflammation leading to tissue damage and ineffective fungal control (31). Deffert *et al*. showed that when a preparation containing the *A. fumigatus* cell wall is introduced, a severe and persistent inflammatory response is induced in a murine knockout model that lacks NADPH oxidase (32). Studies in humans and mice have conclusively demonstrated that infection with *A. nidulans* and *A. fumigatus* induces a hyper-inflammatory response driven by the excessive production of interleukin-1 (31). The treatment for inflammatory complications in patients with CGD and IA includes corticosteroids and other immunosuppressants (31, 33). The 35% of the patients described in our study received steroids; some were part of an anti-inflammatory treatment in conjunction with antimicrobials.

The access to transplants is only available to a minority of patients in Latin America (15). Apart from cost, other limiting factors include lack of expertise in performing transplants on patients with CGD. Among the patients in the present study, 12 underwent SCT, and 50% died during the procedure, five secondary to IA. Countries such as Mexico should take into account the experience gained from other groups: transplantation in CGD should be strongly considered at a younger age and especially if a well-matched donor is available (34, 35). In high-risk patients with CGD, reduced intensity conditioning SCT regimen is safe and effective (36, 37). All patients with CGD should be screened for aspergillosis prior to SCT. Since serum ß-d-glucan and serum galactomannan provide unreliable results, a negative result does not mean that *Aspergillus* spp. infection does not exist; radiological imaging is a relatively more effective indicator of the disease extent (8, 13, 37, 38, 39). Additionally, it is crucial to consider antifungal therapy before and after transplantation regardless of whether the patient is infected by *Aspergillus* (38, 40). Multidrug antifungal therapy is essential for successful transplant outcomes in patients with CGD and fungal infections (13). IA was responsible for an overwhelming majority (69%) of all deaths in this study group of 45 patients. This rate is higher than those reported in other international cohorts (7, 9, 10, 22, 26, 28, 41).

Finally, the early treatment of IA is crucial, which includes empirical combined and aggressive treatments, introduction of new antifungal drugs, measurement of therapeutic levels, and implementation of SCT. These measures will reduce morbidity and mortality in countries with a situation similar to Mexico.

## Materials and methods

Patients with CGD (genetic diagnosis) and IA in Mexico (from seven different hospitals) between January 2005 and September 2024 were included. The study protocol was approved by the Research Committee of the National Institute of Pediatrics (019/2011). Since this was a retrospective study of chart information, informed consent was not required. Attending physicians were contacted and invited to participate by completing a questionnaire on demographic, clinical, microbiological, and therapeutic information related to the IA event(s) of each patient with CGD. Each physician reviewed the medical records of the patients and recorded the requested information. Questionnaires were received electronically. Data were entered into an Excel spreadsheet.

### Statistics

Descriptive statistics were used for the statistical analysis. Data are expressed according to distribution, categorical variables with proportions, and numerical variables as medians (interquartile ranges are in parentheses). Differences between groups were compared using Fisher’s exact test and Mann–Whitney U test. Survival was analyzed by the Kaplan–Meier method, with log-rank test performed for comparisons values of p < 0.05 were considered significant. All statistical analyses were performed using Statistical Package for Social Science (SPSS) software version 25.0 (SPSS, Inc.).

### Definitions

Each IA event was classified as possible, probable, or proven according to the criteria established by the EORTC/MSGERC (17). Patients were considered to have a proven IA event if they satisfied at least one of the following mycological criteria: (1) histopathological, cytopathological, or direct microscopic examination of a specimen obtained by needle aspiration or biopsy in which hyphae specific to *Aspergillus* spp. were observed accompanied by evidence of associated tissue damage (with necessary confirmation by means of culture or polymerase chain reaction). (2) Recovery of *Aspergillus* spp. by culture of a specimen obtained using a sterile procedure from a normally sterile site and a clinically or radiologically abnormal site consistent with an infectious disease process, while excluding bronchoalveolar lavage fluid, cranial sinus cavity specimen, and urine. Probable IA requires the presence of a host factor, a clinical criterion, and a mycological criterion. Cases that met the criteria for host factors and clinical criteria, but not for mycological criteria, were considered as possible IA (17).

The first IA event was defined as the first *Aspergillus* infection. Subsequent events were defined based on the presence of new clinical/radiological data with or without new mycological evidence (direct or indirect) after the completion of antifungal treatment and evidence of clinical/radiological improvement for the first episode (11, 17, 42).

## Data Availability

Data available upon request.
